# Thriving in a hostile world: Insights from the dietary strategy of two allopatric, closely related tepui summit endemic amphibians

**DOI:** 10.1002/ece3.7682

**Published:** 2021-05-27

**Authors:** Philippe J. R. Kok, Tessa L. Broholm, Dietrich Mebs

**Affiliations:** ^1^ Department of Ecology and Vertebrate Zoology University of Łódź Łódź Poland; ^2^ The Natural History Museum London UK; ^3^ Department of Biology Vrije Universiteit Brussel Brussels Belgium; ^4^ Institute of Legal Medicine Goethe University of Frankfurt Frankfurt Germany

**Keywords:** diet, Guyana, OCBIL, Pantepui, tepui, toads

## Abstract

To date, there has been no published investigation on the trophic diversity in any tepui summit vertebrate. In this paper, we analyzed the dietary composition of a tepui summit endemic toad, *Oreophrynella quelchii* from Roraima‐tepui, and compared it with that of *O. nigra* from Kukenán‐tepui, to examine to what extent diet differs between these two sister species across isolated, although neighboring, tepui tops. The digestive tracts of a total of 197 toads were dissected: 111 from *O. quelchii* and 86 from *O. nigra*. The diet composition of *O. quelchii* was relatively diverse, with 13 major prey categories; mites (Acari, 36.5%) and beetles (Coleoptera, 21.0%) numerically dominated its diet. Despite occurring on two different tepui summits, *O. quelchii* and *O. nigra* exhibited a similar diet composition, although in *O. nigra* mites (Acari, 42.4%) and hymenopterans (especially ants, 16.9%) numerically dominated the diet. The present data suggest that tepui summit *Oreophrynella* species are flexible in their diet and are active foragers that also feed on aquatic arthropods, successful strategies in tepui competitive environments.

## Graphical abstract

We analyzed the dietary composition of a tepui summit endemic toad, *Oreophrynella quelchii* from Roraima‐tepui, and compared its diet with that of *O*. *nigra*from Kukenán‐tepui, to examine whether diet differs between these two sister species across isolated, although neighboring, tepui tops. Both species exhibited a similar, surprisingly diverse, diet composition, and results suggest that tepui summit *Oreophrynella* species are flexible in their diet and are active foragers that also feed on aquatic arthropods, successful strategies in tepui competitive environments.

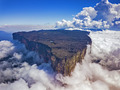

## 1 INTRODUCTION

**FIGURE 1:**
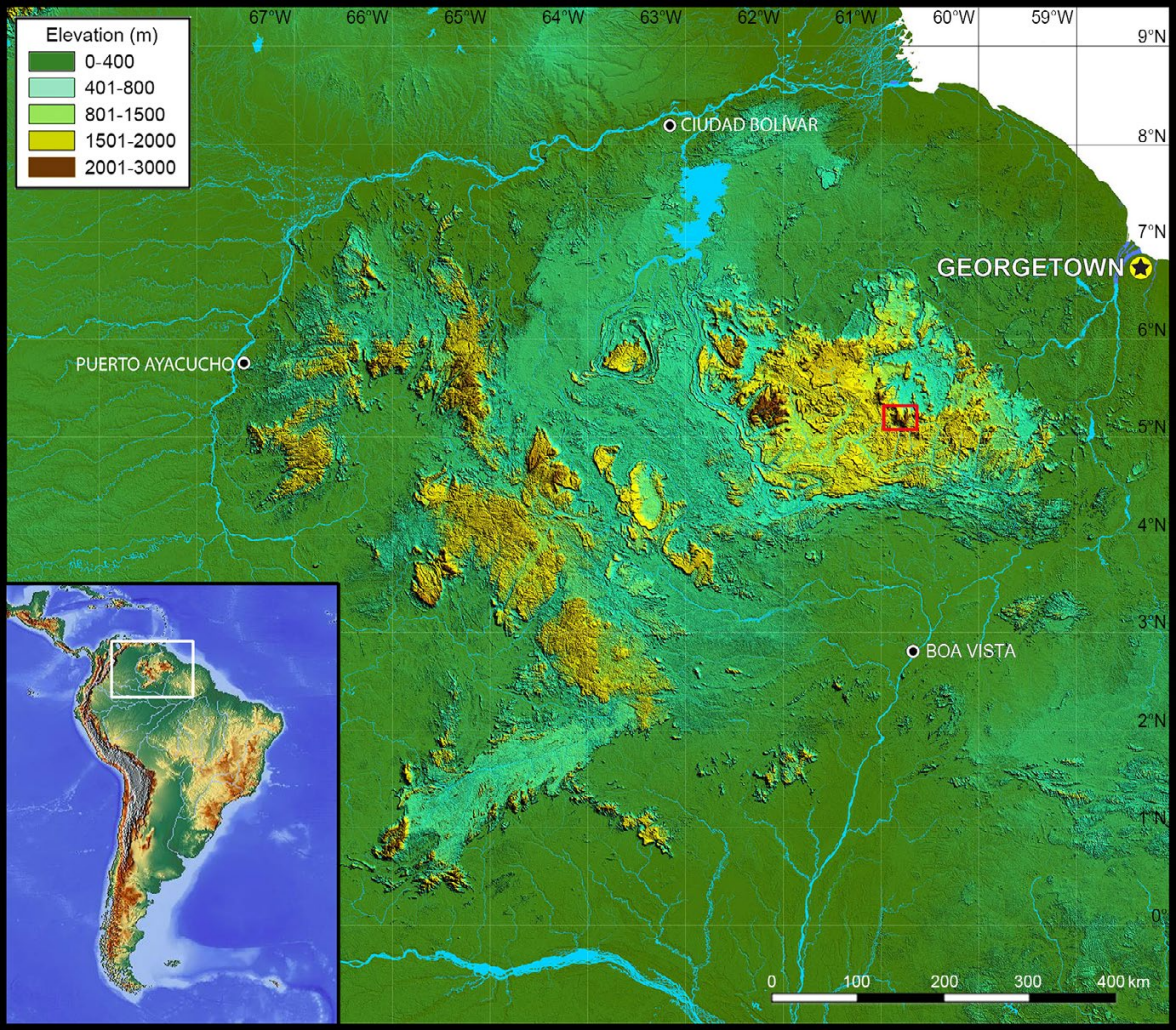
Map of Pantepui and its location in South America. Red rectangle indicates the location of Roraima‐tepui and Kukenán‐tepui as shown in Figure 4

**FIGURE 2:**
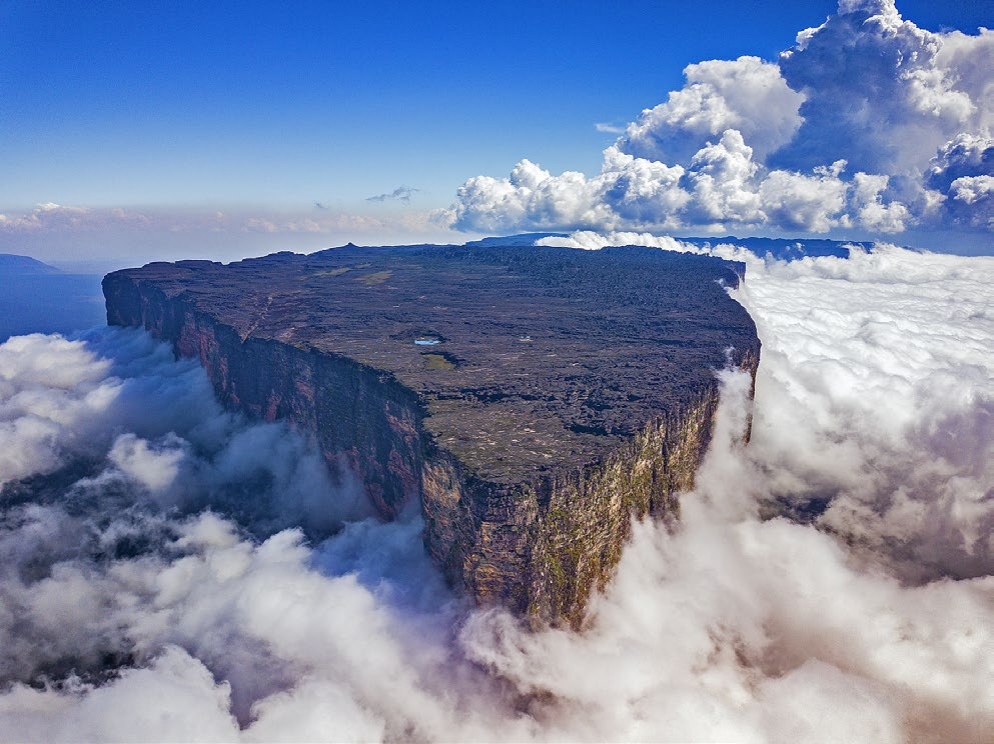
Typical tepui summit. Drone photograph of the “Prow” of Roraima‐tepui (taken on 22 March 2019, facing south) demonstrating the summit's physiographical and ecological isolation from the surrounding uplands

The Guiana Shield highlands in northern South America are one of the most stunning, remote, and least explored biogeographical regions of the world. Often referred to as the “Lost World” based on Arthur Conan Doyle's eponymous fiction novel (Doyle, 1912), this region is called Pantepui (Mayr & Phelps, 1967; Figure 1). Pantepui harbors dozens of isolated Precambrian sandstone tabletop mountains (called “tepuis”; Figure 2) reaching up to ca. 3,000 m elevation and is renowned for its floral and faunal endemism (Berry et al., 1995; Kok, 2013a; McDiarmid & Donnelly, 2005). The high tepui summits are challenging, highly competitive ecosystems that are both physiographically and ecologically isolated from the more fertile surrounding environments. Their vegetation grows on highly acidic, oligotrophic soils and is drastically different from the vegetation of the intervening uplands. Tepui summits are isolated from each other and from the surrounding upland savannah and tropical rainforest by up to 1,000 m high vertical cliffs and face contrasted, particularly hostile climatic conditions (strong cold winds, extreme temperature/hygrometry variation), and high solar and ultraviolet (UV) radiation (McDiarmid & Donnelly, 2005). The summit of Roraima‐tepui (ca. 2,800 m elevation) is characterized by a submicrothermic ombrophilous climate, with heavy rainfall combined with dense cloud and mist formation almost all year, and an average annual air temperature of 8–12°C (Huber, 1995), but we observed that wind chill can drop the temperature to 4–5°C (Kok, unpublished), and minimum air temperatures of 1–2°C have been recorded (Huber, 1995). Rainfall can occur for several consecutive days in the wet season, while severe droughts, with relative hygrometry occasionally dropping to less than 20%, can span over 10 days in the dry season. UV index exceeding 25 has been regularly recorded (Kok, unpublished).

The Pantepui ecosystem is one of the few regions on earth that, since pre‐Pleistocene times, has not experienced surface rejuvenation by either orogenesis or glaciation. Ancient and nutrient‐poor, these landscapes are the so‐called “OCBILs” (Old, Climatically Buffered, Infertile Landscapes) characterized by Hopper (2009). These rare paleosurfaces underwent extended periods of tectonic stasis and subaerial exposure and, according to Hopper's OCBIL theory, their biotic assemblages should differ fundamentally in several traits from floras and faunas evolving in post‐Pleistocene landscapes. In plants, Hopper (2009) listed nutritional specialization as one of the seven predictions derived from the OCBIL theory, but this has never been tested in animals.

During the last decade, many new amphibian and reptile taxa from species (e.g., Carvalho et al., 2010; Fouquet et al., 2015; Gower et al., 2010; Kok, 2010, 2013b; Kok, Bittenbinder, et al., 2018; Kok et al., 2010, 2011, 2015, 2018; Kok & Rivas, 2011; Recoder et al., 2020) to higher‐ranked taxa (e.g., Kok, 2015; Pellegrino et al., 2018; Pinheiro et al., 2019; Sánchez‐Pacheco et al., 2017) have been described in that region, which has been crucial in understanding the evolution of Pantepui amphibians and reptiles. Two pivotal molecular phylogenetic studies (Kok et al., 2012; Salerno et al., 2012) revised an old paradigm that tepui‐summit vertebrate populations have lived in complete isolation for millions of years (McDiarmid & Donnelly, 2005). Both studies support a relatively recent origin of extant tepui summit anuran species, and one of them (Kok et al., 2012) highlights the prevalence of low genetic divergence across most isolated tepui summit amphibian and reptile species/populations.

Although our understanding of phylogenetic evolution on the regional scale is increasing, ecological evolution on a local scale still remains poorly understood in Pantepui. One can assume that tepui summit species responded differently to historical changes because they have a different ecology; thus, the study of their ecology is highly relevant for a better understanding of their evolution on a local scale. However, the natural history and ecology of the tepui‐summit fauna are still mostly *terra incognita*, with only sparse anecdotic data about amphibians, often only briefly discussed in the framework of species descriptions. This is mainly due to the logistical difficulties in performing long‐term field surveys in such extreme and isolated environments. Notable exceptions are a few reports on the reproductive ecology and behavior of tepui summit toads of the genus *Oreophrynella* (Mägdefrau & Mägdefrau, 1994, 2000; McDiarmid & Gorzula, 1989), of *Pristimantis yuruaniensis* (Strabomantidae) and *Stefania riveroi* (Hemiphractidae) from the Eastern Tepui Chain (Mägdefrau & Mägdefrau, 1994), and a recent paper discussing the unexpected predation on *Oreophrynella quelchii* by firefly larvae (Kok et al., 2019). To date, there has been no published investigation on dietary patterns in any tepui summit amphibian species.

Similarly, information about the invertebrate fauna on tepui summits is essentially limited to species descriptions, some of which also provide habitat characteristics. The actual arthropod diversity/abundance of tepui ecosystems is largely unknown. Only a few invertebrate groups have been the subject of more detailed treatments (often taxonomic and/or zoogeographic), such as butterflies (e.g., Viloria & Costa, 2019), scorpions (e.g., Ochoa & Rojas‐Runjaic, 2019), land snails (e.g., Breure, 2019), ants (e.g., Jaffe et al., 1993), and some aquatic insects (e.g., Derka et al., 2019), but these studies provide no or only fragmentary data on the species quantitative abundance and ecology. Furthermore, most of these studies focus on invertebrates that are too large to be preyed upon by *Oreophrynella*. Thus far, food‐web structure on tepui summits remains virtually unstudied.

Toads of the genus *Oreophrynella* (nine species endemic to Pantepui; Kok et al., 2020) are particularly well adapted to the tepui top environment (Kok et al., 2019, 2020; McDiarmid & Gorzula, 1989). Current phylogenetic hypotheses based on molecular data indicate that *Oreophrynella* is placed near the base of the bufonid tree (e.g., Kok et al., 2012; Kok, Ratz, et al., 2018; Van Bocxlaer et al., 2010) in a paraphyletic group of taxa sometimes termed “atelopodid” (e.g., McDiarmid, 1971), with *Atelopus* recovered as its sister clade (Kok, Ratz, et al., 2018). Known closest relatives to *Oreophrynella* occur in significantly different ecosystems than tepui summits (from grasslands and rainforests to paramo). Some dietary data are available for a few of these closely related genera. *Melanophryniscus* currently contains 29 described species distributed in Paraguay, Uruguay, the northern half of Argentina, the inter‐Andean valleys of southern Bolivia, and the coastal lowlands of southern Brazil from ca. sea level to 2,400 m elevation (Frost, 2020). Species in the genus *Melanophryniscus* are reported to primarily feed on ants and mites (e.g., Bokermann, 1967; Bortolini et al., 2013; Duré et al., 2009, 2015; Filipello & Crespo, 1994), although some populations have seemingly more generalized diets with ants being common but not the dominant prey items (e.g., Bonansea & Vaira, 2007). *Atelopus* currently contains 97 described species distributed from Costa Rica to Bolivia and the Guiana Shield from ca. sea level to 3,000 m elevation (Frost, 2020). Durant & Dole (1974) indicated that stomach contents of *A. oxyrhunchus* consisted primarily of beetles, ants, and mites. Toft (1981) stated that *A. varius* is a “pronounced ant specialist”, whereas González et al. (2012) reported ants and beetles as the most consumed preys in *A. cruciger*, both with high frequency of occurrence (87.5% and 80.7%, respectively). *Osornophryne* currently contains 11 described species distributed in the Cordillera Central in Colombia to the central Andes of Ecuador between 2,700 and 3,700 m elevation (Frost, 2020). In *Osornophryne*, Vanegas‐Guerrero et al. (2016) stated that *O. percrassa* is a generalist feeder, as also suggested for other species in the genus (e.g., Gluesenkamp, 1995; Gluesenkamp & Acosta, 2001; Gluesenkamp & Guayasamin, 2008). Methodology and sample size differ significantly across these studies making them difficult to compare in terms of diet specialization/generalization.

Foraging strategies in amphibians span between “sit‐and‐wait” predators (usually when preys are mobile and frequently encountered), and “active” predators (usually when preys are encountered infrequently, and displacements are necessary to increase the probability of prey detection; Huey & Pianka, 1981). Foraging strategies are thus triggered by prey identity and environmental conditions, and it has been shown that predators are able to modify their foraging strategies accordingly (Hodar et al., 2006; Perry, 1999; Scharf et al., 2006).

**FIGURE 3:**
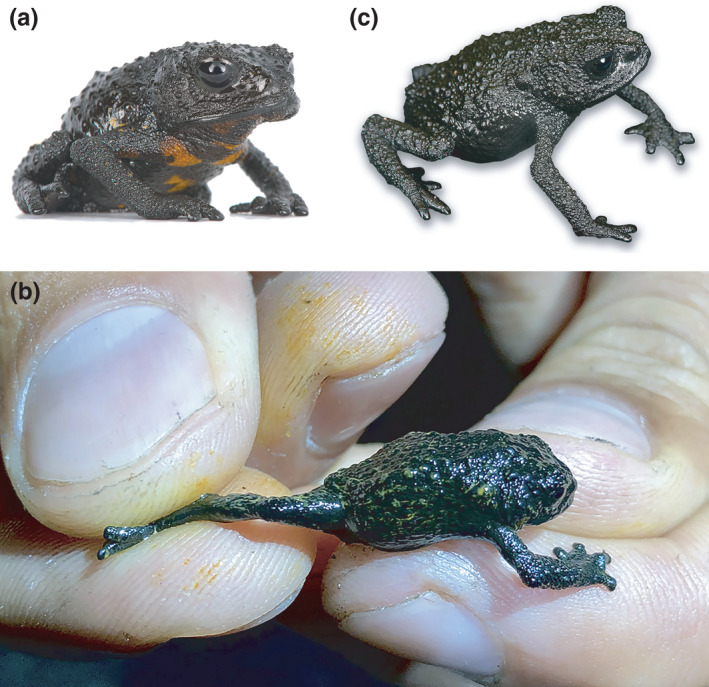
Toad species under study. (a) *Oreophrynella quelchii* from Roraima‐tepui. (b) *O. quelchii* from Roraima‐tepui secreting sticky yellow fluid. (c) *O*. *nigra* from Kukenán‐tepui

*Oreophrynella quelchii* (Figure 3a) is only known from the summit of two neighboring tepuis, Roraima‐tepui (Venezuela‐Guyana‐Brazil; max elevation ca. 2,800 m, summit area ca. 35 km^2^) and Wei‐Assipu‐tepui (Guyana‐Brazil; max elevation ca. 2,260 m, summit area ca. 3 km^2^). The species is reported to occur in high numbers on the summit of Roraima‐tepui (Kok et al., 2019; Kok, unpublished), but is listed as Vulnerable (VU) by the IUCN Red List of Threatened Species since it notably has a very restricted range and is expected to be prone to decline due to global climate change (IUCN SSC Amphibian Specialist Group, 2020). These small toads (16.2–29.8 mm snout‐vent length in adults) secrete a sticky yellow fluid when molested (Figure 3b). Dietary composition and its numerous implications, from spatial ecology to patterns of potential alkaloid sequestration, could be one of the factors behind demographic features in *Oreophrynella*. Thriving in a hostile environment predicts increased diet flexibility (e.g., Blondel & Bourlière, 1979), but one of the OCBIL’s predictions (Hopper, 2009; see above) instead suggests some dietary specialization.

In order to clarify this potential paradox, the main aims of this study were to (1) characterize the trophic diversity of *Oreophrynella quelchii* on the summit of Roraima‐tepui; and (2) compare the diet of *O. quelchii* with that of the closely related, similarly sized, *O. nigra* (16.4–24.7 mm snout‐vent length in adults) living on a close neighboring tepui summit.

## 2 MATERIALS AND METHODS

### 2.1 Study sites and sampling

**FIGURE 4:**
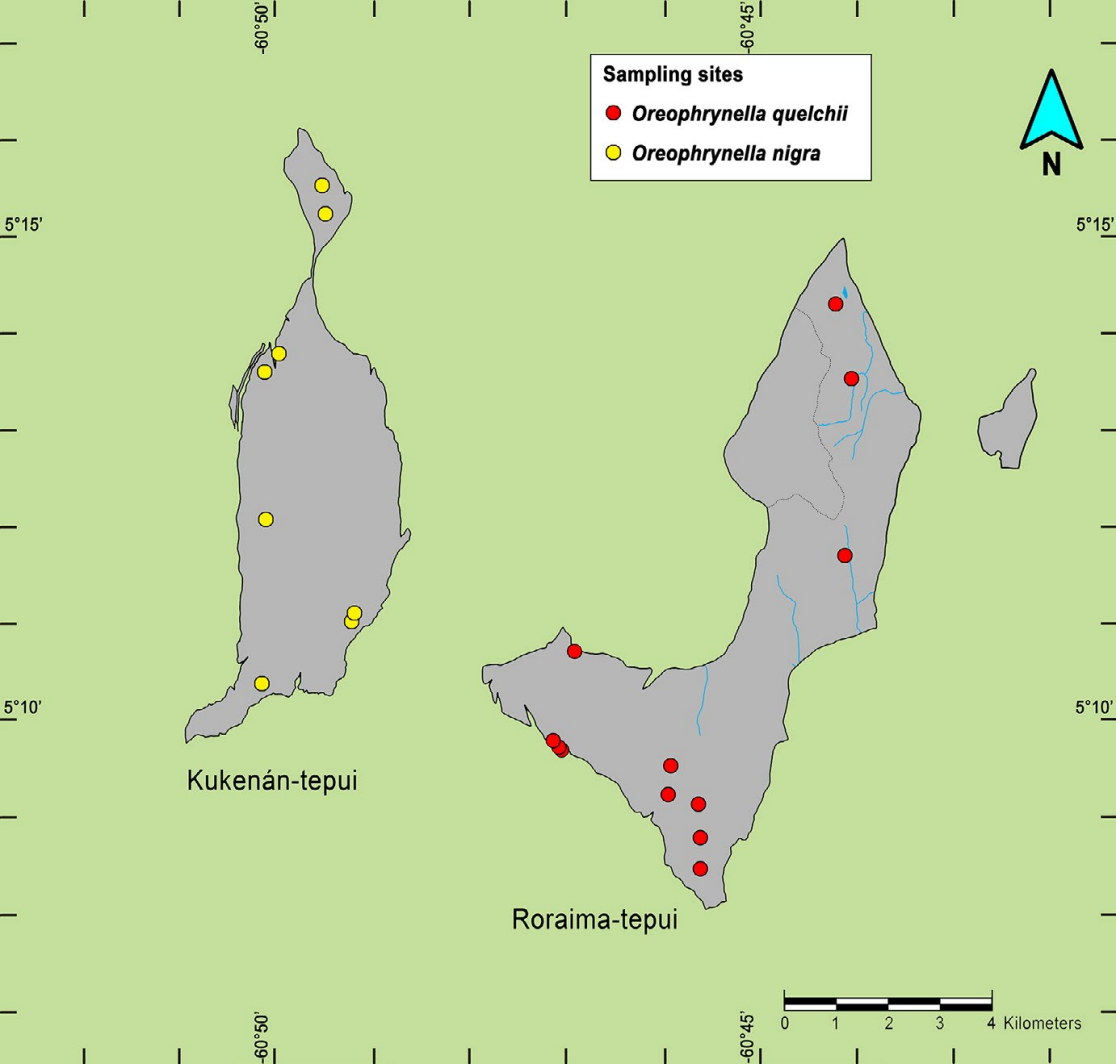
Sketch map of Roraima‐tepui and Kukenán‐tepui. Sampling localities are depicted for *Oreophrynella quelchii* and *O*. *nigra*

The present study was conducted on the summit of two neighboring tepuis located in the Eastern Tepui Chain (sensu McDiarmid & Donnelly, 2005; Figures 1 and 4): Roraima‐tepui (max elevation ca. 2,800 m; summit area ca. 35 km^2^) and Kukenán‐tepui (max elevation ca. 2,650 m; summit area ca. 21 km^2^). The closest linear distance between their summits is ca. 2,600 m. Their rocky surfaces are highly isolated from each other by ca. 400–700 m high vertical cliffs and large stretches of upland savannah and tropical rainforest on their slopes and between their bases.

Specimens from both tepui summits were collected by hand during the wet season (June‐August) at multiple localities across the entire summits (Figure 4), in the framework of a comparative population genetic study (Kok, in progress). We dissected the digestive tract of 111 individuals of *Oreophrynella quelchii* from Roraima‐tepui (Figure 3a) and of 86 individuals of *O*. *nigra* from Kukenán‐tepui (Figure 3c). Gut contents were placed in tubes containing 99% ethanol for preservation.

### 2.2 Dietary analysis

Gut contents were sorted, dried, and mounted on an aluminum holder, sputtered with gold, and analyzed with a Hitachi S‐4500 scanning electron microscope at an acceleration voltage of 5 kV (cold‐field emission electron source). Scanning electron microscopy (*SEM*) images were edited in Adobe Photoshop^®^ without altering their original structure; few micrographs are compositions made of three to four different images because of the larger size of the prey item. Micrographs were sent to invertebrate experts for identification to the lowest taxonomic rank (see Acknowledgments). Individuals containing only unidentifiable matter and debris were discarded from the analysis. For each prey category, in both species of *Oreophrynella*, the frequency of occurrence (%*FO*) was calculated as the number of stomachs that had the same type of prey/total number of stomachs × 100, and the numerical percentage (%*N*) was calculated as the number of individuals of prey category *j*/total number of prey × 100. These analyses were performed on the entire sample and on males and females separately to determine whether sex has an influence on dietary preferences. In both species of *Oreophrynella*, the volume (*V*, expressed in mm^3^) of each prey item was estimated using the formula for an ellipsoid body, as follows: $V=\frac{4\pi }{3}\frac{L}{2}{\left(\frac{W}{2}\right)}^{2}$, where *L* is the maximum length and *W* the maximum width (Hellawell & Abel, 1971). Volume of fragmented prey items that could be identified was estimated using the methods of Hirai and Matsui (2001). Since all prey items were first subjected to *SEM*—the preparation for which sometimes induced distortion of the structural integrity of soft tissues—volume estimates are only best approximations. The volumetric percentage (%*V*) was calculated as the total volume of prey category *j*/total volume of preys × 100. The index of relative importance (*IRI*; Pinkas et al., 1971) was calculated as *IRI* = %*FO* + (%*V* + %*N*), and the proportion of IRI (%*IRI*) was calculated as the IRI of prey category j/total IRI × 100. Standardized niche breadth (*B*_st_) values were calculated using a modified form of the Simpson's index (Colwell & Futuyma, 1971; Levins, 1968), as follows: $B_{\text{st}}=\frac{B‐1}{n‐1}$, where $B=\frac{1}{\sum p_{j}2}$, *p_j_* is the proportion of individuals in prey category *j*, and *n* is the number of prey categories. A low niche breadth value (*B*_st_ closer to zero) is considered to reflect diet specialization on a few prey categories, whereas a high niche breadth value (*B*_st_ closer to one) is considered to reflect more diversity in the diet, thus diet generalism/flexibility (Hurlbert, 1978; Reynolds & Meslow, 1984). As a prerequisite to this analysis, however, it is assumed that the different resources are equally accessible (see Section 4).

Species richness and diversity (Shannon–Wiener index, *H′*) were calculated for each sample and compared between sexes and species using Mann–Whitney–Wilcoxon tests. All statistical analyses were performed in R (R Development Core Team, 2020).

In an attempt to assess prey availability (i.e., proportion of preys in the environment), we additionally collected invertebrates in the habitat of *Oreophrynella quelchii* on the summit of Roraima‐tepui using 10 small pitfall traps (small biodegradable coffee cups a quarter filled with a soapy water solution) installed during 15 consecutive days at a single location (N5°14′ W60°44′) in the dry season. Collected invertebrates were treated as described above for scanning electron microscopy.

## 3 RESULTS

### 3.1 Dietary composition of *Oreophrynella quelchii*

**FIGURE 5:**
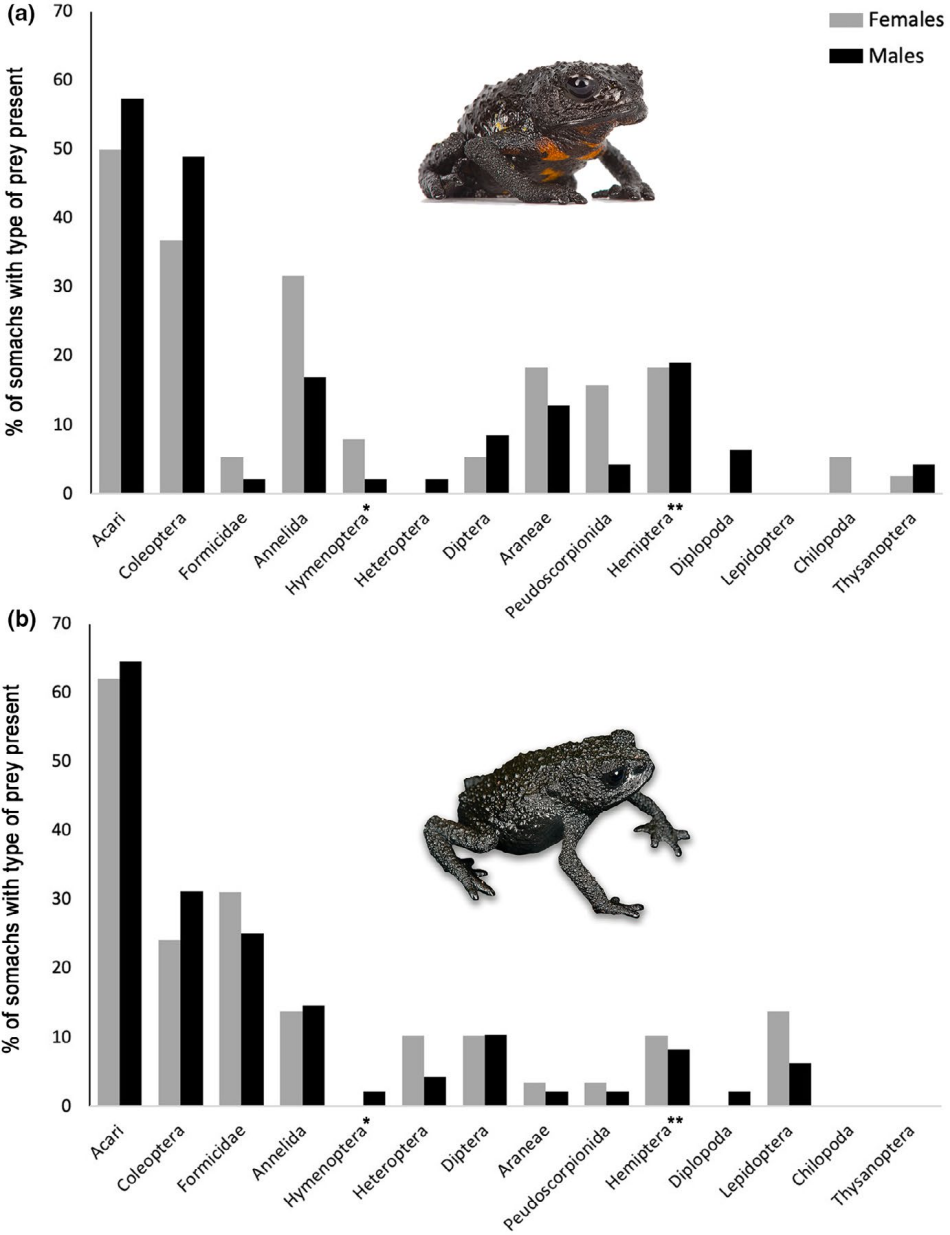
Percentages of identifiable types of prey in males and females. (a) Of *Oreophrynella quelchii* on Roraima‐tepui. (b) Of *O*. *nigra* on Kukenán‐tepui; * = excluding Formicidae, ** = excluding Heteroptera

**FIGURE 6:**
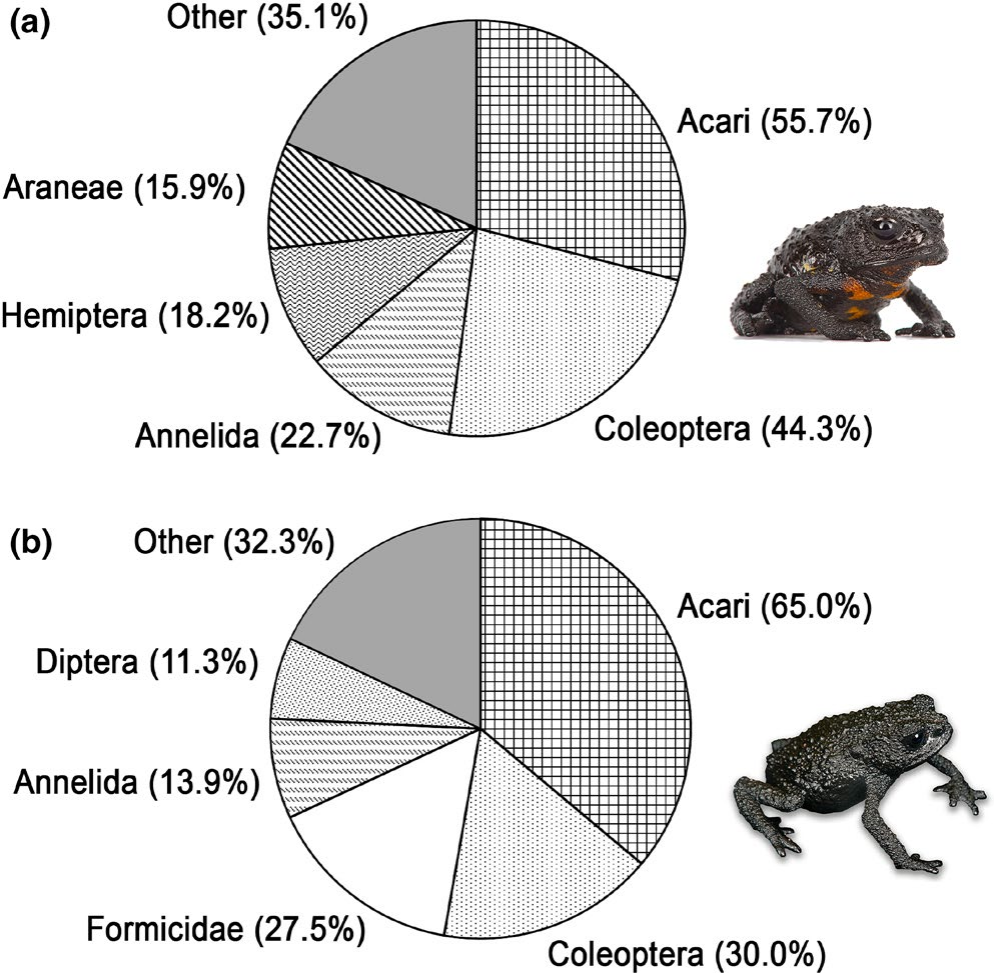
Frequencies of occurrence of important types of prey (i.e., preys that accounted for >10%). (a) In *Oreophrynella quelchii* on Roraima‐tepui. (b) In *O*. *nigra* on Kukenán‐tepui

**FIGURE 7:**
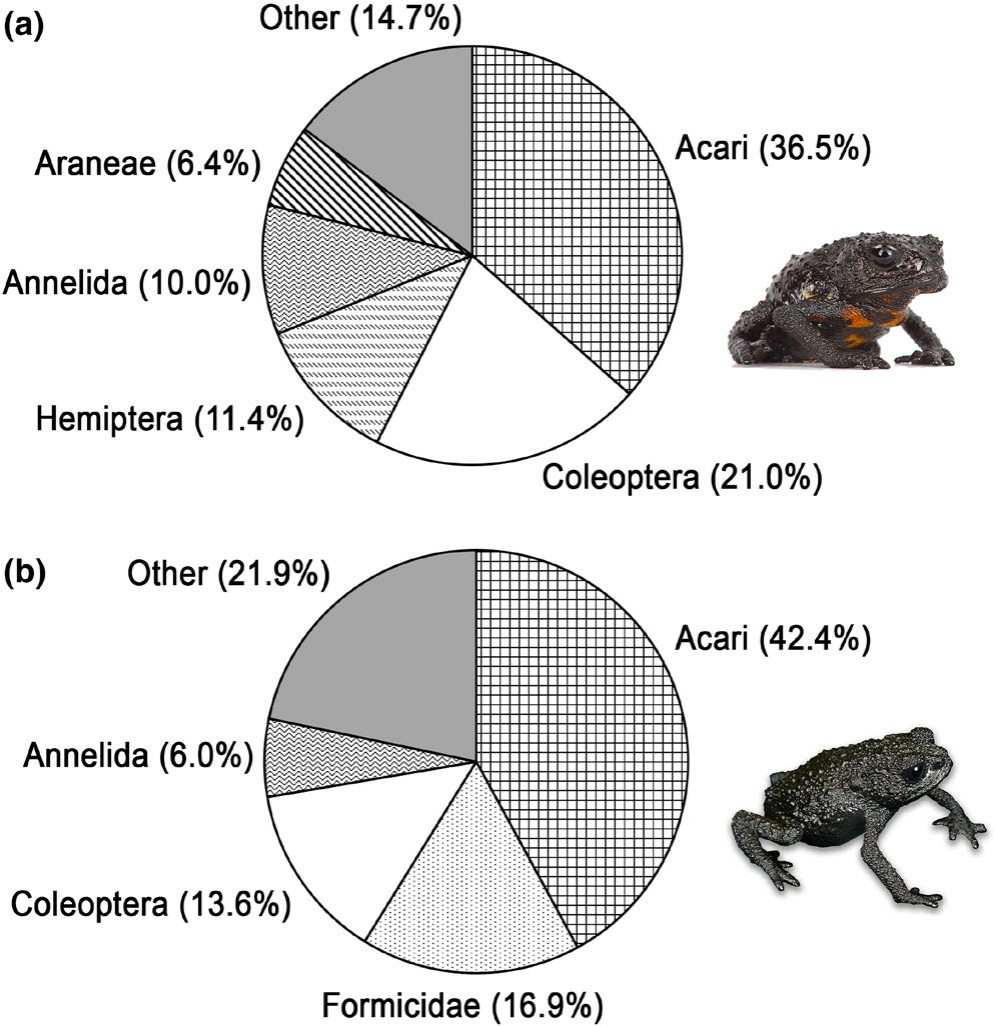
Numerical percentages of important types of prey (i.e., preys that accounted for >10%). (a) In *Oreophrynella quelchii* on Roraima‐tepui. (b) In *O*. *nigra* on Kukenán‐tepui

**FIGURE 8:**
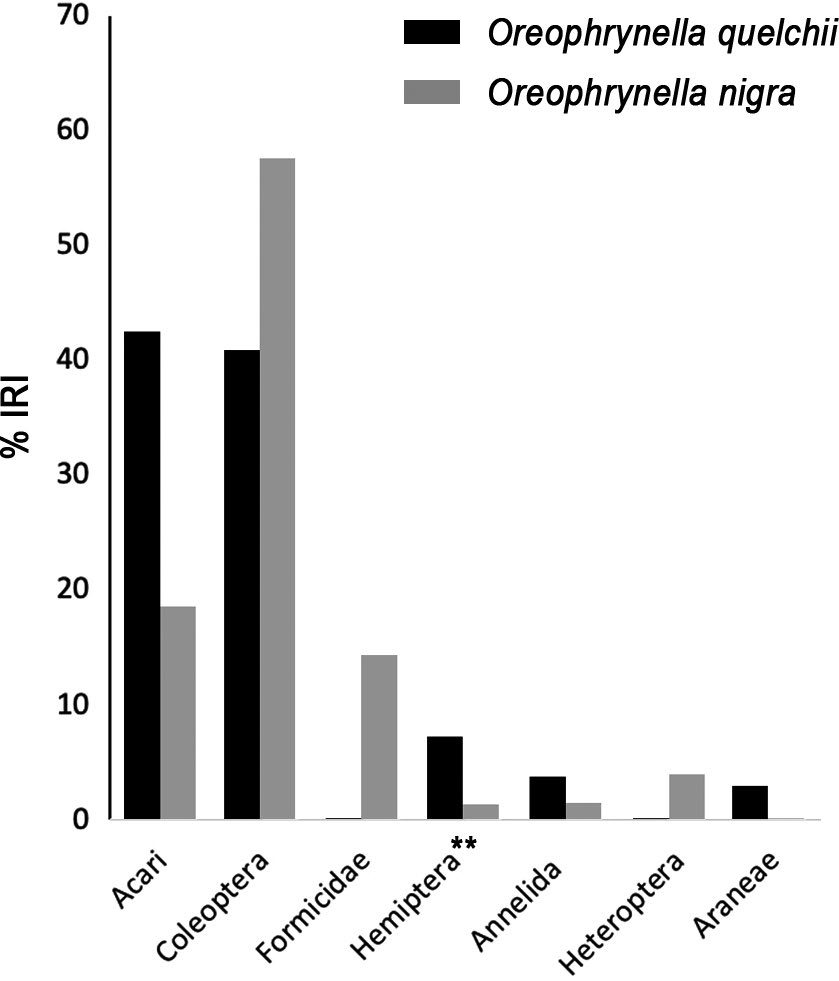
Proportions of index of relative importance (%*IRI*) of seven prey categories with the highest importance (>2.5%) in the diets of *Oreophrynella quelchii* and *O*. *nigra* on Roraima‐tepui and Kukenán‐tepui, respectively; ** = excluding Heteroptera

**FIGURE 9:**
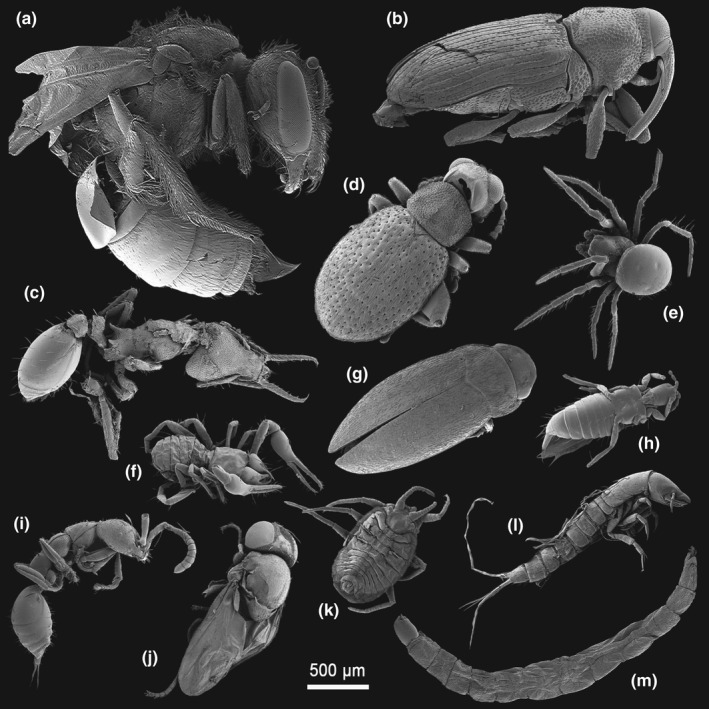
Trophic diversity in *Oreophrynella quelchii*. Selection of scanning electron microscopy images of various arthropods found in the digestive tract of the species. (a) Hymenoptera, Apoidea. (b) Coleoptera, Curculionidae. (c) Hymenoptera, Formicidae, Myrmicinae, *Strumigenys*. (d) Coleoptera, Chrysomelidae, Alticinae. (e) Araneae, Anapidae. (f) Pseudoscorpionida. (g) Coleoptera, Dytiscidae, Hydroporinae, *Tepuidessus breweri*. (h) Thysanoptera. (i) Hymenoptera, Bethylidae. (j) Diptera, Brachycera, Phoridae. (k) Hemiptera, Aphidoidea. (l) Coleoptera, Dytiscidae, Colymbetinae, *Rhantus elegans* (larva). (m) Diptera, Nematocera, Chironomidae (larva)

**FIGURE 10:**
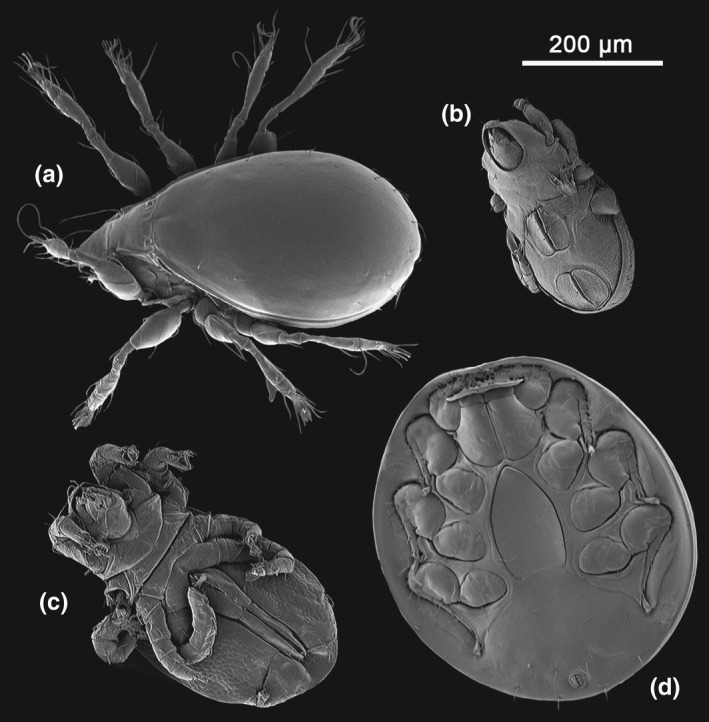
Trophic diversity in *Oreophrynella quelchii*. Selection of scanning electron microscopy images of various Acari found in the digestive tract of the species. (a) Sarcoptiformes, Oribatida, Desmonomata, Oribatulidae. (b) Sarcoptiformes, Oribatida, Desmonomata, Brachypilina. (c) Sarcoptiformes, Oribatida, Desmonomata (tritonymph). (d) Parasitiformes, Mesostigmata, Monogynaspida, Uropodina (ventral face of phoretic deutonymph)

Of 111 digestive tracts dissected, six only were found to be empty (5.4%); 15 (16.7%) contained unidentifiable matter and debris, and were thus discarded from the analysis (Figures 5a, 6a, 7a, 8, 9, 10). The diet composition was surprisingly diverse for a small toad living in such a hostile and supposedly depauperate environment, with 13 major prey categories frequently associated with anurans: Acari, Annelida, Araneae, Chilopoda, Coleoptera, Diplopoda, Diptera, Formicidae, Hemiptera (excluding Heteroptera), Heteroptera, Hymenoptera (excluding Formicidae), Pseudoscorpionida, and Thysanoptera, encompassing mobile and hard‐bodied prey items, and even aquatic arthropods. However, the α‐diversity within each group was low. The mean number of prey items found in stomachs was 2.5 ± 1.4. Prey sizes varied from ca. 0.3 to 5 mm (but see above regarding *SEM* preparation's artifacts). Subclass Acari comprised the largest percentage of identifiable gut contents (*N* = 36.5%, but some of these taxa are likely phoretics and associated with other ingested arthropods [Gagnarli, pers. com.]); the second largest gut content consisted of Coleoptera (*N* = 21%). Acari and Coleoptera also showed the highest frequency (*FO* = 55.7% and *FO* = 44.3%, respectively), and highest proportion of relative importance (%*IRI* = 40.9% and %*IRI* = 42.5%, respectively).

Only two prey items could be identified to the species level, *Tepuidessus breweri* (Figure 9g) and the larva of *Rhantus elegans* (Figure 9l; Dytiscidae). Acari were represented by Uropodina (Parasitiformes, Mesostigmata, Monogynaspida), and by Desmonomata (Sarcoptiformes, Oribatida) such as Oribatulidae and Brachypylina; Coleoptera included a few families, that is, Curculionidae, Chrysomelidae, and Dytiscidae; Hemiptera accounted for 11.4% of the diet (*FO* = 18.2%), represented by the super‐family Aphidoidea; Annelida (Clitellata) accounted for 10.0% of the diet (*FO* = 22.7%, third highest frequency after Acari and Coleoptera); and Araneae, represented by Mygalomorphae and the families Anapidae and Oonopidae, accounted for 6.4% (*FO* = 15.9%). Other prey items consisted of Pseudoscorpionida (*N* = 3.7%; *FO* = 9.1%); Diptera of the families Chironimidae and Phoridae (*N* = 3.2%; *FO* = 6.8%); Thysanoptera (*N* = 1.8%; *FO* = 4.5%); Formicidae (genus *Strumigenys*, *N* = 1.4%; *FO* = 3.4%), while other members of the order Hymenoptera, such as the families Apoidae, Bethylidae, and Diapriidae, accounted for 1.8% (*FO* = 4.5%); Chilopoda (*N* = 0.9%; *FO* = 2.3%); Diplopoda (*N* = 1.4%; *FO* = 3.4%), and Heteroptera (with members of the family Lygaeidae, *N* = 0.5%; *FO* = 1.1%). *B*_st_ = 0.317. Most prey categories were found in both sexes, with Acari and Coleoptera having the highest percentage occurrence in both males and females (Figure 5a). The main dietary difference between sexes in our sample was the consumption of Heteroptera and Diplopoda exclusively by males and of Chilopoda exclusively by females (Figure 5a).

Average taxa diversity in the samples did not statistically significantly differ between males and females (male *H′* = 1.79, female *H′* = 1.74, *w* = 127.5, *p* = .18).

### 3.2 Dietary comparison with *Oreophrynella nigra*

Of the 86 *O*. *nigra* digestive tracts dissected, one only was empty (1.2%), six (4.3%) contained unidentifiable matter and debris, and were thus discarded from the analysis. The diet composition of *Oreophrynella nigra* was almost as diverse as that of *O. quelchii*, with 12 major prey categories: Acari, Annelida, Araneae, Coleoptera, Diplopoda, Diptera, Formicidae, Hemiptera (excluding Heteroptera), Heteroptera, Hymenoptera (excluding Formicidae), Lepidoptera, and Pseudoscorpionida (Figures 5, 6b, 7b and 8). Like in *O. quelchii*, the α‐diversity within each group was low. The mean number of prey items found in stomachs was 2.3 ± 1.6. Prey sizes varied from ca. 0.3 to 3 mm (but see above regarding *SEM* preparation's artifacts). The subclass Acari comprised the largest percentage of identifiable gut contents from *O. nigra* (*N* = 42.4%; *FO* = 65.0%), followed by the family Formicidae (*N* = 16.9%; *FO* = 27.5%, third highest frequency after Acari and Coleoptera). Acari had the highest proportion of relative importance (%*IRI* = 57.6%) followed by Coleoptera (%*IRI* = 18.5%) and Formicidae (%*IRI* = 14.4%). No prey item could be unambiguously identified to the species level. Like in *O. quelchii*, Acari were represented by Uropodina (Parasitiformes, Mesostigmata, Monogynaspida), and by Desmonomata (Sarcoptiformes, Oribatida) such as Oribatulidae and Brachypylina. Two genera of Formicidae were identified (versus one in *O. quelchii*), *Strumigenys* and *Solenopsis*, while other members of the order Hymenoptera, represented by the families Ceraphronidae and Diapriidae, only accounted for 1.1% (*FO* = 1.3%); Coleoptera were represented by members of the families Chrysomelidae and Dytiscidae (*Tepuidessus* sp) and accounted for 13.6% (*FO* = 30.0%, second highest frequency after Acari like in *O. quelchii*). Annelida (Clitellata) accounted for 6.0% of the diet (*FO* = 13.8%) of *O. nigra*. Larvae of Lepidoptera accounted for 4.4% (*FO* = 8.8%), versus no Lepidoptera identified in the gut contents of *O*. *quelchii*; Diptera for 4.9% (*FO* = 11.3%); Hemiptera for 4.4% (*FO* = 8.8%), represented by the super‐family Aphidoidea; Heteroptera (with members of the family Lygaeidae) for 3.3% (*FO* = 6.3%); Pseudoscorpionida for 1.6% (*FO* = 3.8%); Araneae (represented by the family Tetragnathidae) for 1.1% (*FO* = 2.5%); and Diplopoda for 1.1% (*FO* = 1.3%). Unlike *O. quelchii*, no Chilopoda and no Thysanoptera were found to be present in the gut contents. *B*_st_ = 0.266. Most prey categories were found in both sexes, with Acari and Coleoptera having the highest percentage occurrence in males, while in females Acari and Formicidae had the highest percentage occurrence (Figure 5b). The main dietary difference between sexes in our sample was the consumption of Hymenoptera (excluding Formicidae) and Diplopoda exclusively by males (Figure 5b).

Average taxa diversity in the samples did not statistically significantly differ between males and females (male *H′* = 1.77, female *H′* = 1.93, *w* = 114, *p* = .47). No statistical difference was found when populations of *O. nigra* and *O. quelchii* were compared (*O*. *nigra H′* = 1.84, *O*. *quelchii H′* = 1.93, *w* = 88, *p* = .66).

### 3.3 Pitfall trapping

Pitfall traps installed during the dry season caught few taxa compared with the high diversity found in the gut contents of the toads, and we consider our data to be insufficient to estimate how selective *Oreophrynella* are among available preys (see Section 4). Mesofauna captured included mites (Acari), bugs (Heteroptera, e.g., Lygaeidae), small flies (Diptera), water beetles (Coleoptera, Dytiscidae, *Tepuidessus breweri*), and springtails (Collembola).

## 4 DISCUSSION

### 4.1 Dietary flexibility in *Oreophrynella quelchii* and *O*. *nigra*

Population density reflects the balance of recruitment and mortality, which are affected by the availability of resources, predation risk, and optimal foraging behavior. Flexible foraging strategies and diet generalism are thus key to species thriving in hostile environments, like tepui summit endemic toads of the genus *Oreophrynella*. Optimal foraging theory predicts that when resources are scarce optimal foragers include less valuable prey items into their diet and become generalists (Pyke et al., 1977; Stephens & Krebs, 1986). Foraging mode may also affect vagility, and thus spatial ecology, through morphological and physiological (i.e., endurance) adaptions (Toft, 1980), and generalist feeders are reported to be more sedentary (e.g., Toft, 1981). However, characterizing specialists versus generalists is a more complex issue than might first appear, mainly because the degree of specialization is a continuum rather than a rigid dichotomy; such characterization remains thus arbitrary (e.g., Darst et al., 2005). Dietary niche breadth values are frequently used to reflect the degree of diet specialization. Specialists are characterized by low niche breadth values, whereas generalists exhibit high ones (Hurlbert, 1978; Reynolds & Meslow, 1984). However, the use of niche breadth indexes to estimate niche breadth values notably assumes that the spectrum of dietary resources available, and the abundance of each of these resources, are known. While this is the case for large apex predators living in well‐studied ecotypes (see Lyngdoh et al., 2014; O’Donoghue et al., 1998), it rarely occurs for vertebrates that mainly feed on mesofauna, such as many small amphibian species. In these instances, authors sometimes take for granted that the different resources are equally accessible, and their abundance not subjected to interspecific competition for example, which can lead to incorrect results (see Feinsinger et al., 1981). To our knowledge (see also Díaz‐Perez et al., 2020; Rebouças & Solé, 2015; Solé & Rödder, 2010), only a small proportions of the studies on the feeding ecology of anurans include quantitative data on prey availability or abundance that are necessary to elucidate selective feeding [i.e., when prey items are selected disproportionately to their availability, or are proportionally underrepresented (Lechowiz, 1982)] and opportunistic feeding [i.e., when similar proportions of prey items are found in the diet and in the environment (Chesson, 1978; Lechowiz, 1982)]. Yet quantitative data on prey availability and abundance are not free of bias as, for example, abundance of preys can vary spatially and over time, some ingested preys can be phoretic (see below), or harder to collect in the environment. Overall, determining whether species (or populations, or even individuals of a same species) are specialized by choice or by circumstances is intricate. In the present case, pitfall trapping did not record enough data to estimate how selective *Oreophrynella* are among available preys, mostly because the trap sampling was much less diverse compared with the taxa found in the gut contents. The few micro‐arthropods collected in pitfall traps correspond to those consumed by *Oreophrynella*, except for Collembola, suggesting that the toads may avoid this taxon. Our trapping method may have been more effective at collecting more mobile arthropods (a known issue, see Ausden, 1996; Solé & Rödder, 2010), but active foraging behavior could also explain the discrepancy between the diversity of preys found in the traps as compared to that found in the toad's gut contents. Although we recovered low standard niche breadth values (*B*_st_ < 0.4, suggesting specialization, e.g., Novakowski et al., 2008), our overall results indicate a relatively high trophic diversity in *O. quelchii* and *O. nigra*, and the number of invertebrate taxa found in the digestive tracts of both species did not highlight any significant bias toward a specific group. No group had *FO* and/or *IRI* ≥ 80%, both common features in specialist frog species (e.g., Ahmad Sah et al., 2019; Díaz‐Perez et al., 2020) that would indicate a trend toward specialization (Blondel & Bourlière, 1979; Cody, 1974; Rolando, 1990). The numerical dominance of common small terrestrial invertebrates, such as mites and beetles in the diet of these small toads, is not surprising. The limited consumption of some groups, such as flying insects or even ants, is likely the result of limited availability of these preys to the toads. Some (possibly a substantial proportion) of the Acari taxa found in the gut contents are phoretic parasites that are associated with other ingested arthropods, and thus not voluntarily ingested by the toads. The magnitude of this bias is currently difficult to evaluate because of our insufficient knowledge of the taxonomy of these parasites on tepui tops. *Oreophrynella quelchii* and *O. nigra* exhibited similarly diverse diets, despite their presence on different tepui summits (although %*IRI* differed, see Figure 8), with Acari and Coleoptera being the most frequent prey items. These prey items contain a high proportion of chitin and are probably costly to digest (Simon & Toft, 1991), especially in a cold environment such as on high tepui summits. This may partly explain the unusual behavior of these toads basking on warm dry ground under high UV/solar radiation (Kok, pers. obs.), which most likely increases metabolism and digestion (e.g., Seymour, 1972). In both species, only males were found to feed on Diplopoda. No significant differences were found between sexes in both species (see Section 3). Both species feed on aquatic arthropods, which confirms field observations of *O. quelchii* sometimes catching prey at the surface of small puddles, and gives another evidence for their foraging plasticity. We also observed individuals of *O. quelchii* eating even when disturbed, for example, when manipulated during photographic sessions. We suggest that the present data support the hypothesis of *Oreophrynella* species being mostly opportunistic/foraging predators that catch any prey of suitable size, an effective strategy in these extreme, competitive environments, and possibly one of the reasons for their success on tepui tops. Nutritional specialization in plants was listed by Hopper (2009) as one of the seven predictions derived from the OCBIL theory. According to our results, this prediction does not stand for amphibians in the area.

### 4.2 Diet comparison with related genera

Diet comparison between *Oreophrynella quelchii*/*O. nigra* and closely related genera is made difficult by substantial differences in sample sizes and methodological approaches across available studies. Moreover, genera that are known to be closely related to *Oreophrynella* live in very different macro‐ and microhabitats. Although studies suggest some *Atelopus* species tend to specialize on ants (e.g., Toft, 1981), others indicate a generalized diet but with a significant bias toward ants and beetles (%*FO* ≥ 80% in these two groups, e.g., González et al., 2012), which suggests a more specialized diet than in *Oreophrynella*. Both diet specialists and diet generalists seem to occur in the genus *Melanophryniscus* (see Bonansea & Vaira, 2007), while *Osornophryne* species are reported as generalist feeders (see Gluesenkamp, 1995; Gluesenkamp & Acosta, 2001; Gluesenkamp & Guayasamin, 2008; Vanegas‐Guerrero et al., 2016), like our results suggest for two species of *Oreophrynella*. Interestingly, both *Oreophrynella* and *Osornophryne* occur at high elevation in challenging and competitive ecosystems (tepui summits for *O. quelchii* and *O. nigra*, the Andes for *Osornophryne*), where increased diet flexibility is likely a better strategy than specialization (e.g., Blondel & Bourlière, 1979; Stephens & Krebs, 1986).

### 4.3 Insights into trophic webs atop tepui summits

As much remains unknown about tepui summit invertebrate fauna, the generalist diets of *Oreophrynella quelchii* and *O*. *nigra* gave more insight into the composition and trophic webs that occur atop tepui summits. The analysis of the diets of *O. quelchii* and *O. nigra* revealed the presence of the ant genus *Strumigenys* (Figure 9c) on the summits of Roraima‐tepui and Kukenán‐tepui, while previous entomological surveys (e.g., Jaffe et al., 1993) only reported the genus *Solenopsis* on these tepuis. We also illustrate for the first time the previously unknown larva of *Rhantus elegans*, a dytiscid reported to be endemic to the summit of Roraima‐tepui (Figure 9l), and demonstrate the presence of the dytiscid genus *Tepuidessus*, likely *T. breweri*, on the summit of Kukenán‐tepui. These examples illustrate how the arthropod diversity of tepui ecosystems is still poorly understood and how the study of stomach contents of anurans could reveal the presence of invertebrate species that are not detected using classical trapping methods in complex microhabitats such as those on tepui summits [see Rabeling et al. (2016) who discovered a new ant species from the stomach of *Oophaga sylvatica* (Dendrobatidae)]. Although *Oreophrynella quelchii* is probably an opportunistic feeder, a notable exception is the avoidance of predatory larvae as noted both through absence in stomach contents and observations. *Oreophrynella quelchii* is predated by venomous firefly larvae (Coleoptera, Lampyridae) on the summit of Roraima‐tepui (Kok et al., 2019). Although early instars of these larvae are within the range of the prey sizes usually consumed, they were never found in the toad's gut contents. This confirms observations on *O. quelchii* individuals actively avoiding firefly larvae when kept together in a small container in the field laboratory. These captive toads never attempted to feed on the firefly larvae, which suggests that they are able to differentiate between firefly larvae and other potential prey items, even at night and even if these larvae do not glow (Kok et al., 2019; Kok, pers. obs.).

### 4.4 Skin secretions in *Oreophrynella*

Tetrodotoxin and/or alkaloids have been reported in the skin of early‐branching “atelopodid” toads, notably in genera closely related to *Oreophrynella*, such as *Atelopus* (Mebs et al., 1995; reported as sister to *Oreophrynella*, see Kok, Ratz, et al., 2018) and *Melanophryniscus* (Mebs et al., 2005). These defensive skin secretions are presumably formed from dietary precursors (Daly et al., 1997) and have not been detected in skin extracts from *Oreophrynella* species collected on the summits of Tramen‐tepui, Kukenán‐tepui, and Yuruaní‐tepui in the early nineties (Mebs et al., 1995). Our preliminary analyses of the skin secretion of *O. quelchii* from the summit of Roraima‐tepui did not detect typical toad bufadienolides, amines, or alkaloids. It remains to be determined if the apparent lack of alkaloids in *Oreophrynella* is linked to a lack (or loss) of the ability to sequester alkaloids from dietary arthropods. In this case, there would be no strong evolutionary pressure toward a specialized diet, allowing these toads to be more flexible feeders, as our data suggest. The chemical nature of the yellow fluid secreted by *O. quelchii* (possibly endogenously biosynthesized peptides/proteins) still needs to be analyzed provided that more material becomes available. The potential toxicity of these secretions combined with the dietary plasticity of these small toads could partly explain their success in the highly contrasted environmental conditions of tepui summits.

## CONFLICT OF INTEREST

The authors declare no conflict of interest.

## AUTHOR CONTRIBUTION

**Philippe J. R. Kok:** Conceptualization (lead); Formal analysis (equal); Funding acquisition (lead); Investigation (lead); Methodology (equal); Supervision (lead); Writing‐original draft (lead); Writing‐review & editing (lead). **Tessa L**. **Broholm:** Formal analysis (equal); Writing‐original draft (supporting); Writing‐review & editing (supporting). **Dietrich** **Mebs:** Formal analysis (equal); Methodology (equal); Writing‐original draft (equal); Writing‐review & editing (equal).

## Data Availability

Data that support the findings of this study are available from Dryad at https://doi.org/10.5061/dryad.sf7m0cg6c.
